# Usher Syndrome: Genetics and Molecular Links of Hearing Loss and Directions for Therapy

**DOI:** 10.3389/fgene.2020.565216

**Published:** 2020-10-22

**Authors:** Meg Whatley, Abbie Francis, Zi Ying Ng, Xin Ee Khoh, Marcus D. Atlas, Rodney J. Dilley, Elaine Y. M. Wong

**Affiliations:** ^1^ Ear Science Institute Australia, Nedlands, WA, Australia; ^2^ Emergency Medicine, The University of Western Australia, Nedlands, WA, Australia; ^3^ School of Human Sciences, The University of Western Australia, Nedlands, WA, Australia; ^4^ Ear Sciences Centre, The University of Western Australia, Nedlands, WA, Australia; ^5^ Centre for Cell Therapy and Regenerative Medicine, The University of Western Australia, Perth, WA, Australia; ^6^ School of Pharmacy and Biomedical Sciences, Faculty of Health Sciences, Curtin University, Bentley, WA, Australia

**Keywords:** hearing loss, Usher syndrome, hair cell, stereocilia, retinitis pigmentosa, photoreceptor, inner ear

## Abstract

Usher syndrome (USH) is an autosomal recessive (AR) disorder that permanently and severely affects the senses of hearing, vision, and balance. Three clinically distinct types of USH have been identified, decreasing in severity from Type 1 to 3, with symptoms of sensorineural hearing loss (SNHL), retinitis pigmentosa (RP), and vestibular dysfunction. There are currently nine confirmed and two suspected USH-causative genes, and a further three candidate loci have been mapped. The proteins encoded by these genes form complexes that play critical roles in the development and maintenance of cellular structures within the inner ear and retina, which have minimal capacity for repair or regeneration. In the cochlea, stereocilia are located on the apical surface of inner ear hair cells (HC) and are responsible for transducing mechanical stimuli from sound pressure waves into chemical signals. These signals are then detected by the auditory nerve fibers, transmitted to the brain and interpreted as sound. Disease-causing mutations in USH genes can destabilize the tip links that bind the stereocilia to each other, and cause defects in protein trafficking and stereocilia bundle morphology, thereby inhibiting mechanosensory transduction. This review summarizes the current knowledge on Usher syndrome with a particular emphasis on mutations in USH genes, USH protein structures, and functional analyses in animal models. Currently, there is no cure for USH. However, the genetic therapies that are rapidly developing will benefit from this compilation of detailed genetic information to identify the most effective strategies for restoring functional USH proteins.

## Introduction

### Usher Syndrome

Usher syndrome (USH) is an autosomal recessive (AR) disorder characterized by sensorineural hearing loss (SNHL), vision loss due to retinitis pigmentosa (RP), and vestibular dysfunction (Geleoc and Holt, 2014; Mathur and Yang, 2015). USH has an estimated global prevalence of between 4 and 17 cases per 100,000 individuals, and accounts for approximately 50% of all hereditary deaf-blindness cases and 3–6% of all childhood hearing loss (HL) cases (Boughman et al., 1983; Fortnum et al., 2001; Kimberling et al., 2010; Lenarduzzi et al., 2015). The syndrome was first described in 1858, by German ophthalmologist Albrecht Von Graefe, in three siblings with simultaneous congenital deafness and RP (von Graefe, 1858; Boughman et al., 1983). USH was later named after Charles Usher, a Scottish ophthalmologist who established its heritability in 1914 based on 69 cases (Usher, 1914).

Usher syndrome is genetically heterogeneous with nine causative genes confirmed, two suspected, and a further three candidate loci having been mapped. USH has been considered to be a monogenic, genetically heterogeneous disease from the very beginning. However, several studies have shown the digenic inheritance of deafness caused by mutations in USH genes and USH modifier PDZD7 in mice and humans (Zheng et al., 2005, 2012; Ebermann et al., 2011; Bonnet et al., 2016). Zheng et al. (2005, 2012) demonstrated that compound heterozygous *Cdh23*/*Pcdh15*; *Myo7a*/*Ush1g*; *Myo7a*/*Cdh23*; *Myo7a*/*Pcdh15* mice exhibited HL and disorganized hair-cell stereocilia. Inner ear hair cells (HC) have no capacity for regeneration after birth. Once damaged or when these cells begin to deteriorate, which can occur during development, HL progresses and is irreversible (Bermingham-McDonogh and Reh, 2011; Franco and Malgrange, 2017).

An understanding of the USH protein functions and interactions within the inner ear will allow for functional domain analysis and therefore the prediction of mutation pathogenicity within USH genes. This knowledge is critical for the successful development of SNHL treatment in USH patients, since almost all modern therapies, including gene, cell, and drug therapies, rely on the thorough understanding of the molecular basis of the disease. Here, we summarize the current literature on the USH genes and their protein structure, function, and localization, and elucidate the disease mechanisms underlying known USH-causing mutations.

### Inner Ear Structure and Function

**Figure 1:**
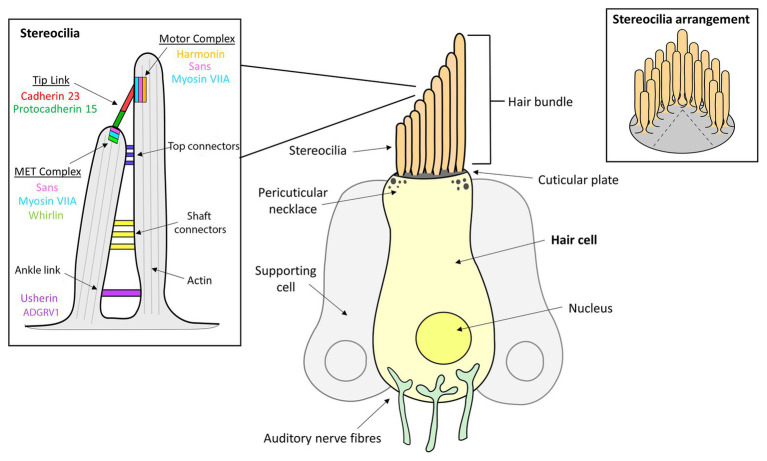
Inner ear hair cell (HC) structure showing the locations of several Usher syndrome (USH) proteins. The apical side of each hair cell expresses a bundle of highly-organized, actin-filled stereocilia, in which the mechanotransduction takes place. The stereocilia are held together by transient ankle links at their base, horizontal shaft links along their length, horizontal tip links near the top and diagonal tip links at their apices.

The organ of Corti is the hearing sensory organ located within the cochlea of the human inner ear and contains approximately 16,000 HC (Schwander et al., 2012; Mathur and Yang, 2015). The HC are arranged as one row of inner HC (IHC) and three rows of outer HC (OHC; Mathur and Yang, 2015; Franco and Malgrange, 2017). All HC have a bundle of approximately 100 actin-rich microvilli, called stereocilia, on their apical surface that are arranged in an inverted V shape, with the length of these projections decreasing stepwise from the tallest stereocilium adjacent to the kinocilium (Pickles et al., 1984; Kachar et al., 2000; Sakaguchi et al., 2009). The single tubulin-filled kinocilium composed of a 9 + 2 microtubule structure is located on the apical surface of HC (Sakaguchi et al., 2009). Upon maturation of the mammalian cochlea, the ankle links and most lateral links are eventually lost and the kinocilium is reabsorbed such that mature mammalian cochlear HC lack kinocilia (Figure 1; Hudspeth and Jacobs, 1979; Verpy et al., 2011). Five different types of supporting cells are organized in rows along the organ of Corti, namely: (1) Hensen’s cells; (2) Deiters’ cells; (3) Pillar cells; (4) Inner phalangeal cells; and (5) Border cells. Supporting cells in mature sensory epithelia preserve the structural integrity of the sensory organs, modulate homeostasis, and maintain the extracellular matrices that enable hair cell mechanotransduction (Slepecky et al., 1995). The sound transduction process occurs at these stereocilia, where mechanical stimuli are converted by a mechanotransduction process into chemical signals, which are then transmitted *via* auditory nerve fibers through to the brain (Tilney et al., 1980).

In the developing cochlea, hair cell development and maturation proceed in two perpendicular gradients: from the base to apex; and from the medial to lateral aspects of the cochlea. During hair bundle development, the kinocilium derives from the primary cilium, migrates from the center to the lateral edge of the hair cell apex. After that, microvilli around the kinocilium elongate to form stereocilia of graded heights. Stereocilia undergo further row-specific, differential outgrowth, eventually forming a hair bundle with a staircase organization (Kelly and Chen, 2009; Wong et al., 2016). The planar polarity and staircase-like pattern of the hair bundle are essential for the mechanoelectrical transduction (MET) function of inner ear sensory cells.

The stereocilia are angled toward the kinocilium and are anchored together through a series of extracellular protein filaments that interconnect and link them, the molecular composition of which change during developmental stages (Ahmed et al., 2006; Sakaguchi et al., 2009; Indzhykulian et al., 2013). Tip links are protein complexes that connect the tip of the shorter stereocilium to their taller neighbor and are a critical component of mechanotransduction since they are involved in transmitting physical force to the mechanotransduction channels located at the tip of the stereocilia (Assad et al., 1991; Sakaguchi et al., 2009; Maeda et al., 2017). Tip links are comprised primarily of protocadherin 15 and cadherin 23, and interact with a number of other USH proteins that both anchor and stiffen the links (Corns et al., 2018). Additionally, stereocilia are joined by horizontal or lateral links that are involved in maintaining the organization and structure of the bundle, as well as the stiffness required for mechanotransduction to occur (Hackney and Furness, 2013). Specifically, there are top and shaft horizontal connectors, which have slightly different composition and function. During early development, the bundles also contain transient ankle links and kinociliary links, involved in interconnecting the base of each of the stereocilia and the kinocilium to the bundle, respectively (Hackney and Furness, 2013).

#### Mechanoelectrical Transduction

The mechanoelectrical transduction process in the cochlea is essential for the conversion of mechanical signals into chemical responses, to communicate sound signals to the brain (Grati and Kachar, 2011; Blanco-Sanchez et al., 2018). MET relies on the appropriate organization of actin-based stereocilia to open the potassium channels and depolarize the cells (Corns et al., 2018). Mutations causing abnormal development of these HC structures can disrupt the MET process, contributing to SNHL and deafness (Blanco-Sanchez et al., 2018).

The resting potential of HC is approximately −60 mV (Bracho and Budelli, 1978; Gillespie and Muller, 2009). Upon opening of the mechanotransduction channels, the cell depolarizes toward 0 mV, which initiates a signaling cascade (Gillespie and Muller, 2009). Sound waves entering the ear cause displacement of the hair bundle, which results in the tip links “pulling” open the mechanotransduction ion channels, specifically those for potassium and calcium. The ion channels are located at the lower end of the tip link, and their opening causes an inward positive current and depolarization of the cell (Powers et al., 2017). This depolarization activates the release of neurotransmitters at the base of the HC, which excite the auditory nerve fibers and result in sound perception (Gillespie and Muller, 2009; Powers et al., 2017).

## Usher Subtypes and Genes

Three clinical subtypes of USH have been defined – Type 1, Type 2, and Type 3 – based upon the presence, severity, and progression of auditory, visual, and vestibular symptoms. Type 1 (USH1) accounts for approximately one third of USH cases and is the most severe form, with profound SNHL and vestibular dysfunction from birth, as well as progressive RP (Smith et al., 1992b, 1994; Weil et al., 1995; Hope et al., 1997). Type 2 (USH2) is the most common subtype, accounting for more than half of all USH cases worldwide (Ng et al., 2019). USH2 patients present with moderate-to-severe SNHL, normal vestibular function, and RP that begins generally during puberty. Type 3 (USH3) is the rarest form, accounting for approximately 2% of all cases (Jouret et al., 2019). Patients with USH3 have normal physiology at birth, with variable levels of hearing, vision, and vestibular deterioration over time (Ng et al., 2019). HL occurs prior to visual symptoms in all three subtypes (Jouret et al., 2019). Fourteen USH loci have been mapped so far, with nine for USH1, three for USH2, two for USH3, as well as one USH modifier and one atypical USH gene (Table 1; Mathur and Yang, 2015). However, some cases are not attributed to these genes and are instead categorized as atypical USH (Otterstedde et al., 2001).

**Table 1:** The subtypes of USH and their known associated genes and proteins.

Subtype	Locus	Location	Gene	Protein	Expression in inner ear
USH1	USH1A	Withdrawn			
	USH1B	11q13.5	MYO7A	Myosin VIIa	throughout hair cells HC
	USH1C	11p15.1	USH1C	Harmonin	Upper tip link density UTLD, HC synapses
	USH1D	10q22.1	CDH23	Cadherin 23	Development: transient lateral links and kinociliary links, HC synapses Postnatal: tip links, HC synapses
	USH1E	21q21	USH1E	n/a	
	USH1F	10q21.1	PCDH15	Protocadherin 15	Development: transient lateral links and kinociliary links, HC synapses Postnatal: tip links, HC synapses
	USH1G	17q25.1	USH1G	SANS	Upper tip link density UTLD
	USH1H	15q22-23	USH1H	n/a	
	USH1J	15q25.1	CIB2/DFNB48	CIB2	stereocilia, esp. tip
	USH1K	10p11.21-q21.2	USH1K	n/a	
USH2	USH2A	1q41	USH2A	Usherin	Development: ankle links, HC synapses Postnatal: hair bundles, HC synapses
	USH2B	Withdrawn			
	USH2C	5q14.3-21.3	GPR98 (also known as ADGRV1)	ADGRV1	Development: ankle links, HC synapses Postnatal: HC synapses
	USH2D	9q32-q34	DFNB31 (also known as *WHRN*)	Whirlin	Development: ankle links, HC synapses Postnatal: stereociliary tip, HC synapses
USH3	USH3A	3q21-q25	CLRN-1	Clarin-1	hair bundles, spiral ganglion, HC synapses
	USH3B	5q31.3	HARS	HARS	supporting cells
n/a	n/a	n/a	PDZD7	PDZD7	Development: ankle links

### USH Type 1

In addition to congenital SNHL and vestibular dysfunction, USH1 patients suffer from tunnel vision within the first decade of life and are often considered legally blind by mid-life due to RP (Lenassi et al., 2014; Ng et al., 2019). USH1 is further subtyped based on the specific causative gene involved. To date, nine causative loci have been mapped for USH1 with mutations in *MYO7A* mutations being the most common cause of USH1, followed by mutations in *CDH23* and *PCDH15* (Ahmed et al., 2001; Jouret et al., 2019). USH1A was first described and mapped to the long arm of chromosome 14 at position 32.1 (14q32.1) in France (Kaplan et al., 1992). The USH1A subtype has now been withdrawn as the disease-causing mutations identified by Kaplan et al. (1992) were later found to be located at other USH type 1 loci (Larget-Piet et al., 1994; Gerber et al., 2006). Although the loci of *USH1E*, *USH1H*, and *USH1K* have been mapped, the resulting proteins have not yet been identified (Chaib et al., 1997; Ahmed et al., 2009; Jaworek et al., 2012).

#### Usher Type 1B: Myosin VIIA

Mutations in *MYO7A* are associated with USH1B and account for approximately 21% of all USH cases and 50% of USH1 cases (Weil et al., 1995; Roux et al., 2006; Jaijo et al., 2007; Yoshimura et al., 2016; Jouret et al., 2019). Myosin VIIa encodes an actin-binding motor protein that facilitates the movement of intracellular proteins along the actin filaments of HC stereocilia, thereby aiding the migration of proteins into their appropriate positions (Maravillas-Montero and Santos-Argumedo, 2012). Myosin VIIa also participates in the development of actin bundles in HC (Heissler and Manstein, 2012; Sato et al., 2017). Therefore, it contributes to the spatial and temporal organization of proteins in HC, and defects in this mechanism can significantly influence stereocilia formation and affect cell function (Kros et al., 2002; Jaijo et al., 2007). In the cochlea, myosin VIIa is expressed throughout the stereocilia and cytoplasm of both IHC and OHC, particularly at the cuticular plate and peri-cuticular necklace region of stereocilia (Hasson et al., 1995; Heissler and Manstein, 2012; Sato et al., 2017). Abnormal myosin VIIa expression in the inner ear will affect protein trafficking and results in major functional abnormalities of the cochlea HC and their stereocilia, which interferes with sound transduction and therefore leads to SNHL or USH (Kros et al., 2002; Jaijo et al., 2007).

##### Structure

**Figure 2:**
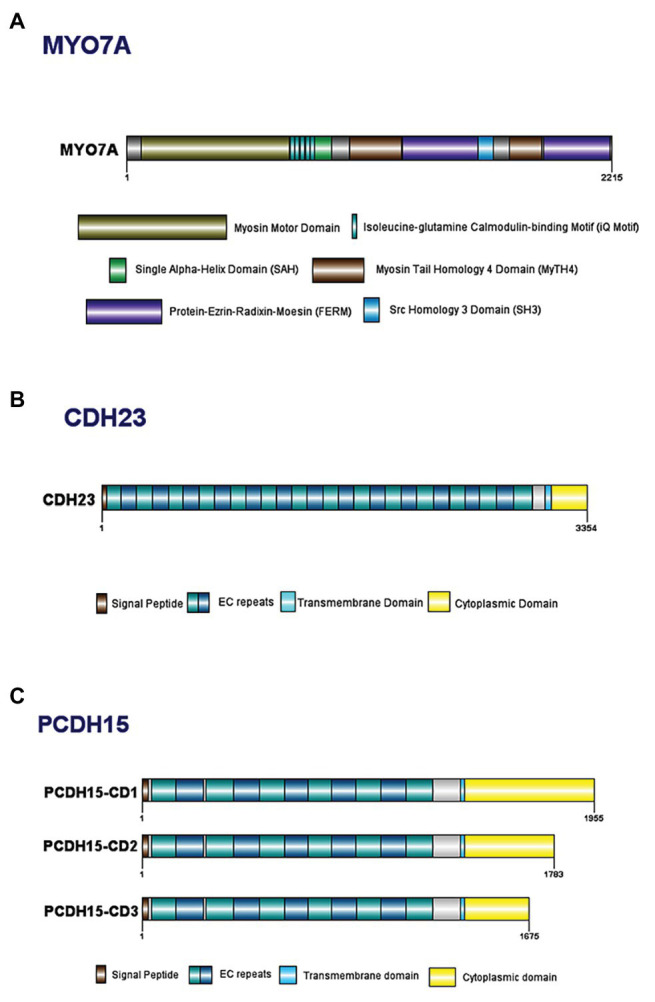
Domain structure of key Usher Type I proteins and their major known isoforms: **(A)** myosin VIIa; **(B)** cadherin 23; and **(C)** protocadherin 15.

The *MYO7A* gene spans 120 kilobases (kb) of genomic DNA, contains 49 exons, and has a 7.4 kb transcribed region (Kimberling and Smith, 1992; Smith et al., 1992a; Guilford et al., 1994; Kelley et al., 1997). *MYO7A* encodes a 254 kDa, 2215 amino acid (aa) protein comprised of a highly conserved N-terminal motor domain containing actin and ATP binding sites (Figure 2A; Kelley et al., 1997; Cheng et al., 2018). The motor domain binds actin filaments and uses energy generated from ATP hydrolysis to move along the filament, allowing intracellular movement of organelles (Udovichenko et al., 2002; Woolner and Bement, 2009). This domain can be monomeric or dimeric, depending on dimerization with the USH1G protein, SANS (Kiehart et al., 2004; Yu et al., 2017). A recently discovered 56 aa SRC Homology 3 (SH3)-like subdomain has been identified near the motor domain (Maravillas-Montero and Santos-Argumedo, 2012). Next is the neck region, with five isoleucine-glutamine calmodulin-binding (IQ) motifs that interact with calmodulin light chains and act as a lever to allow the motor domain to move (Todorov et al., 2001; Udovichenko et al., 2002; Maravillas-Montero and Santos-Argumedo, 2012). There is a predicted single alpha-helix (SAH) domain that is thought to be a divergent type of SAH as it lacks the dense networks of charged amino acids usually typical of SAH domains (Simm et al., 2017). Lastly is a C-terminal tail region with two sets of Myosin Tail Homology 4 (MyTH4) and Protein-Ezrin-Radixin-Moesin (FERM) domains separated by an SH3 domain (Kelley et al., 1997; Weck et al., 2016). The main function of the tail region is to serve as an anchor to position the motor domain such that it can interact with actin filaments (Inoue and Ikebe, 2003; Li et al., 2017).

##### Mutations

Various USH mutations are located throughout the *MYO7A* gene; however, no hotspot mutations have yet been identified (Jaijo et al., 2007). Approximately, 60% of USH1B causative mutations result in a prematurely truncated protein and about 36% are missense mutations (Le Quesne Stabej et al., 2012). As well as USH, two forms of non-syndromic HL and deafness, DFNB2 and DFNA11, are associated with *MYO7A* mutations (Tamagawa et al., 1996; Weil et al., 1997; Liu et al., 1998). DFNB2 is a rare AR disorder, resulting in severe progressive hearing dysfunction with either congenital or early childhood-onset (Hildebrand et al., 2010). DFNA11 is an autosomal dominant disorder, showing progressive HL with variable vestibular dysfunction (Tamagawa et al., 1996; Venkatesh et al., 2015). The phenotype, either syndromic or isolated HL, is dependent on the location and nature of the mutation within the *MYO7A* gene, and is the result of allelic heterogeneity (Weil et al., 1997; Venkatesh et al., 2015).

##### Animal Models

*Shaker-1* mice have normal early development, followed by progressive hair bundle disorganization and abnormal auditory responses (Gibson et al., 1995; Mburu et al., 1997; Self et al., 1998). *Shaker-1* mice exhibit HL or deafness, circling behavior and a head tossing phenotype (Mburu et al., 1997; Self et al., 1998). Some *Shaker-1* models have naturally occurring mutations (e.g., p.R502P and p.R241P), while others have induced mutations within the motor domain or the MyTH/FERM domains of myosin VIIa (Mburu et al., 1997; Self et al., 1998). *Headbanger* mice have the mutation p.I178F and exhibit HL, abnormal balance and a head tossing phenotype resulting in vestibular and cochlea hair bundle defects (Rhodes et al., 2004). *Ewaso* mice have the mutation p.I487N and have abnormal vestibular hair cells (Calabro et al., 2019). *Dumbo* mice have the mutation p.F947I and exhibit disorganized cochlear hair cells during development (Miller et al., 2012). The function of the *MYO7A* ortholog in zebrafish is similar to that of both humans and mice, and zebrafish with mutations in these genes are known as *mariners* (Ernest et al., 2000). *Mariners* are the equivalent to the *Shaker-1* mice models and exhibit vestibular dysfunction, as evidenced by circling behaviors, and have disorganized stereocilia and atypical physiological responses to sound (Ernest et al., 2000; Williams, 2008). *Mariner* zebrafish have mutations affecting the polypeptide domain, motor domain, IQ motifs, and FERM domains (Ernest et al., 2000). Interestingly, 80% of known USH1B-causing missense mutations affect the amino acids that are conserved in the zebrafish myosin VIIa cDNA, further supporting this model to observe specific mutations (Ernest et al., 2000).

#### Usher Type 1C: Harmonin

The causative gene for the USH type 1C phenotype is *USH1C*, which codes for the protein harmonin. Harmonin plays a critical role in the auditory, visual, and vestibular systems (Verpy et al., 2000). Mutations in *USH1C* cause symptoms consistent with USH (Verpy et al., 2000). USH1C accounts for approximately 15% of USH1 cases and 2% of all USH cases (Verpy et al., 2000; Le Quesne Stabej et al., 2012; Jouret et al., 2019).

##### Structure

Harmonin is a PDZ domain-containing scaffold protein, named after the Greek word for assembly (harmonia) for the role it plays in binding the USH complex (Verpy et al., 2000). There are three PDZ1 domains, two coiled-coil domains, and a Proline/Serine/Threonine (PST) region. PDZ domains bind to specific subcellular domains of other proteins and are involved in the organization and anchoring of protein interactomes (Ponting et al., 1997; Fanning and Anderson, 1999). In the inner ear HC, the three harmonin PDZ domains bind to several USH proteins, including myosin VIIa and cadherin 23, and act as a central scaffold for the bundling and localization of the USH protein complex (Verpy et al., 2000; Boeda et al., 2002; Adato et al., 2005b).

There are at least 10 alternatively spliced isoforms of harmonin, which are divided into three subclasses (a, b, and c), seven of which are alternatively spliced exons only expressed in the inner ear (A-F, G/G; Verpy et al., 2000). This suggests mutations that selectively affect these isoforms would cause non-syndromic recessive deafness rather than USH due to the absence of retinal expression and therefore phenotype (Toriello and Smith, 2013). All isoforms contain the first two PDZ domains and the first coiled-coil domain (Verpy et al., 2000). Subcellular localization of the harmonin isoforms varies during developmental stages (Adato et al., 2005b). Unlike the c isoforms, which are the shortest and only contain the common regions shared by all isoform subclasses, the a and b isoforms all have a third PDZ domain. The b isoforms are the longest and contain a second coiled-coil domain, and a PST region that is responsible for calcium-independent binding of actin. This enables these b isoforms to act as an F-actin-bundling-protein and play a role in stabilization of the stereocilia actin fibers (Verpy et al., 2000; Boeda et al., 2002).

##### Mutations

The c.496+1G>A mutation in *USH1C* occurs at a higher frequency than all other USH1 mutations, with more than 9% of USH1 mutant alleles carrying the mutation in one study cohort (Le Quesne Stabej et al., 2012), presenting a possible hotspot. In the Acadian population of Canada, a c.216G>A founder mutation in *USH1C* accounts for almost all cases of USH1C (Ouyang et al., 2003; Ebermann et al., 2007a). This mutation introduces a cryptic 5' splice site into exon 3, which is used favorably over the original 5' splice site, causing a transcriptional frameshift, premature stop codon, and truncated harmonin protein (Ebermann et al., 2007a). Additionally, mutations in *USH1C* can also cause non-syndromic recessive deafness type 18 (DFNB18; Ahmed et al., 2002).

##### Animal Models

*Deaf circler* (*dfcr*) mice and *deaf circler 2 Jackson* (*dfcr-2J*) mice carry mutations in the *Ush1c* gene, the mouse ortholog of *USH1C*, and were developed to improve understanding of the function of harmonin in the inner ear and eye. These mice exhibit morphological abnormalities of the inner ear HC stereocilia and a phenotype of SNHL and vestibular dysfunction, as evidenced by circling behaviors (Johnson et al., 2003). The *dfcr* mice carry a large 12.8 kb intragenic deletion that removes eight exons from the transcript, resulting in the loss of both coiled-coil domains and the PST region (Johnson et al., 2003; Xu et al., 2009). The *dfcr-2J* mice carry a single base deletion that causes a frameshift and results in the inappropriate translation of 38 aa and a truncated protein. The mutations of both models alter the subclass b isoforms, which is expressed in both ear and eye, while only the *dfcr* mutation disrupts the harmonin isoforms a and c (Johnson et al., 2003).

An *Ush1c216AA* knockin mouse model was generated specifically to study the Acadian USH1C founder mutation in exon 3. These mice are homozygous for a 35 bp deletion that mimics that of USH1C patients (Lentz et al., 2007, 2010). In contrast to the other models, *Ush1c216AA* knockin mice exhibit SNHL, vestibular dysfunction, and clear retinal degeneration (Lentz et al., 2007, 2010; Vijayakumar et al., 2017).

#### Usher Type 1D: Cadherin 23

The USH type 1D (USH1D) causative gene, *CDH23*, encodes cadherin 23 (Bolz et al., 2001; Bork et al., 2001). Cadherin 23 is a member of the cadherin superfamily of calcium-dependent cell adhesion molecules. It is a non-classical cadherin, as characterized by its long extracellular domain that is required for the development and correct morphology of the inner ear HC bundles and, along with protocadherin 15, forms the tip links between stereocilia (Kazmierczak et al., 2007; Colas-Algora and Millan, 2019). *CDH23* mRNA is highly expressed in tip links and lateral links of inner ear HC stereocilia (Lagziel et al., 2009).

##### Structure

The human *CDH23* gene spans ~420 kb of genomic DNA and is comprised of 70 exons, that encode a predicted 3354 aa protein. Cadherin 23 has a 5' untranslated region followed by 27 extracellular cadherin (EC) repeat domains, a transmembrane domain, and a unique cytoplasmic domain (CD; Figure 2B; Di Palma et al., 2001b; Oshima et al., 2008). Each EC repeat contains cadherin-specific aa motifs required for cadherin dimerization and calcium binding (Ozawa et al., 1990; Suzuki, 1996; Rowlands et al., 2000). The EC domains form dimers on the cell surface, which bind to dimers on a different cell, thereby linking the cells together. In the absence of calcium, the EC regions are weakened and unable to bind, ultimately resulting in breakage of tip links (Di Palma et al., 2001b). The transmembrane domain consists of single chain glycoprotein repeats and acts as a bridge between the cytoplasmic and extracellular domains and an anchor to fix the cadherin to the cell surface (Shapiro and Weis, 2009). Lastly, the CD contains two PDZ-binding motifs (PBM) that facilitate interactions with other hair bundle proteins that contain PDZ domains, such as harmonin (Boeda et al., 2002; Siemens et al., 2002; Lagziel et al., 2009).

Alternative splicing produces *CDH23* isoforms of three different classes (A, B, and C), which are differentially expressed based on tissue location (Lagziel et al., 2009). Each isoform class has two subtypes, which differ based on their inclusion of exon 69 encodes 35 aa, and was frequently referred to as exon 68 by earlier papers that did not classify the exon in the 5'-untranslated region as exon 1 (Takahashi et al., 2016). The A isoforms contain all 27 EC repeats and are the only isoforms expressed in the inner ear and retina (Takahashi et al., 2016). The B and C isoforms lack many key regions, for example, the C isoforms are entirely cytoplasmic, making them unable to serve a cell-cell adhesion role (Takahashi et al., 2016).

##### Mutations

Mutations in *CDH23* are associated with USH1D, accounting for approximately 6% of all USH cases, as well as non-syndromic AR deafness type 12 (DFNB12), and age-related HL (Di Palma et al., 2001b; Jouret et al., 2019). Over 200 pathogenic *CDH23* mutations have been identified. Typically, nonsense, frameshift or splice-site mutations that result in truncated non-functional forms of cadherin 23 are associated with USH1D, whereas missense mutations are more often associated with DFNB12 (Bork et al., 2001; Astuto et al., 2002). The spectrum of mutations in the global population shows regional variation, with Japanese HL patients having a particularly high frequency of *CDH23* mutations (Mizutari et al., 2015).

##### Animal Models

The *waltzer* mouse carries mutations in *Cdh23* that results in disorganization of the HC stereocilia (Di Palma et al., 2001a). *Waltzer* mice are characterized by deafness and a head shaking and circling phenotype indicative of vestibular dysfunction (Wilson et al., 2001). Mutations in the EC repeats can cause disruptions in the ability of calcium to bind, thereby inhibiting cell-cell interactions.

The zebrafish ortholog of *CDH23* is expressed in the inner ear HC, brain, olfactory organ, and retina, and the protein has been localized to the distal tips of hair bundles, especially of the tallest stereocilia next to the kinocilium (Sollner et al., 2004). The zebrafish model with *cdh23* mutations, *sputnik*, has reduced or absent MET, likely due to the observed detachment of stereocilia bundles from kinocilium and splayed stereocilia (Nicolson et al., 1998; Seiler and Nicolson, 1999; Sollner et al., 2004).

#### Usher Type 1F: Protocadherin 15

Mutations in the *PCDH15* gene result in one of the most severe forms of Usher syndrome, type 1F (USH1F) which was first described in 1997 (Wayne, 1997; Ahmed et al., 2001). The *PCDH15* gene is located on the long arm of chromosome 10 at position 21.1 (10q21.1; Yan and Liu, 2010). *PCDH15* contains 1 Mb of DNA and has an open reading frame of 7,021 bp that codes for the cell-cell adhesion protein protocadherin 15 (Ahmed et al., 2001; Le Guédard et al., 2007). Protocadherin 15 is a non-classical cadherin with a long extracellular region (Ahmed et al., 2001, 2003). Within the inner ear HC, protocadherin 15 is expressed throughout the kinociliary link, transient lateral link, tip link, and synapse, as well as the supporting cells and spiral ganglion of the inner ear (Alagramam et al., 2001b; Ahmed et al., 2006; Mathur and Yang, 2015).

Protocadherin 15 is essential for normal function and development of the mammalian inner ear, as it forms the lower portion of the tip link and binds to cadherin 23 (Alagramam et al., 2001b, 2011; Indzhykulian et al., 2013; Perreault-Micale et al., 2014). The structure of the tip link is transient, with two protocadherin 15 proteins binding as the link during development (Indzhykulian et al., 2013). Evidence suggests that individual protocadherin 15 monomers could function as a gating spring, converting displacement of the HC bundle into the mechanical forces required to open the transduction channels. Although the tip link bundle is too stiff to act as a spring, individual protocadherin molecules have low stiffness, are unstable when under high tension, and display a large range of reversible unfolding when Ca^2+^ levels are reduced. Whether the tip link cadherins are folded during normal hearing remains unknown (Bartsch et al., 2019).

##### Structure

All protocadherin 15 isoforms are comprised of a N-terminal signal peptide domain, an extracellular domain containing tandemly repeated EC motifs, a transmembrane domain, and one of three C-terminal CDs (Figure 2C; Ahmed et al., 2008). The protocadherin 15 isoforms contain EC repeats of ~100 aa in length, connected to each other *via* ~10 aa linker regions (Bolz et al., 2002). These repeats are involved in mediating calcium-dependent binding between cadherin proteins (Bolz et al., 2002; Reiners et al., 2005a). For example, the two most N-terminal cadherin repeats bind to the extracellular region of cadherin 23 to form an extracellular link (Ahmed et al., 2006; Kazmierczak et al., 2007; Nie et al., 2016).

There are four alternative isoforms of protocadherin 15 (CD1, CD2, CD3, and secreted) that vary based on their CDs (Ahmed et al., 2008). The largest protocadherin 15 isoform (CD1) contains 32 protein-coding exons and one non-coding exon that translates the longest protein with 1955 aa, CD2 and CC3 contain 1783 aa and 1675 aa, respectively (Figure 2C; Le Guédard et al., 2007). Each of these CDs contains two highly conserved, proline-rich regions that act as binding sites for proteins containing WW and SH3 domains (Sudol, 1998; Alagramam et al., 2001b; Le Guédard et al., 2007). WW and SH3 domains play a role in the construction of signaling complexes and interact with proteins that are often involved in regulating actin filament polymerization; therefore conservation of these amino acid sequences within protocadherin 15 suggest the protein is involved in the regulation of stereocilia planar polarity (Sudol, 1998; Alagramam et al., 2001b). Each of the three isoforms has a different spatiotemporal expression in both developing and mature HC; therefore, it is likely that each of the isoforms plays a specific role in the HC structure or function (Ahmed et al., 2006, 2008; Le Guédard et al., 2007).

##### Mutations

A large number of disease-causing *PCDH15* mutations are splice sites or frameshifts, which result in a truncated or non-functional protein (Le Quesne Stabej et al., 2012). Approximately, 30% of mutations identified within *PCDH15* are large deletions, most likely a direct cause of the large size of the gene and low number of protein coding regions (Roux et al., 2006; Le Guédard et al., 2007). Although mutations in the *PCDH15* gene account for only 3% of USH cases, an increased incidence of USH1F is observed in the American Ashkenazi Jewish population (Perreault-Micale et al., 2014; Jouret et al., 2019). Mutations in *PCDH15* are also associated with non-syndromic deafness type 23 (DFNB23; Ahmed et al., 2003).

##### Animal Models

The *Ames waltzer* mouse carries mutations in *Pcdh15*, and exhibit similar symptoms to human USH1F patients, such as deafness, and show circling behaviors indicative of vestibular dysfunction (Ahmed et al., 2001; Alagramam et al., 2001a; Haywood-Watson et al., 2006). Cochlea and vestibular HC of *Ames waltzer* mice have disorganized stereocilia bundles and kinocilium, abnormal cell polarization and the OHC lack the V-shaped stereocilia configuration essential for normal MET (Pickles et al., 1984; Alagramam et al., 2001a; Kikkawa et al., 2008). Interestingly, isoform B seems to be the most significant *Pcdh15* isoform for hearing. Mice deficient in isoform B are deaf and have abnormal hair bundles, while mice deficient in either isoform A or C have normal hair bundles and hearing (Kikkawa et al., 2008; Webb et al., 2011).

*Noddy* mice harbor a homozygous p.I108N missense mutation within the first EC repeat and exhibit head-bobbing and circling behaviors, as well as no response to stimulus at any of the frequencies tested, indicative of a complete loss of inner ear function (Geng et al., 2013). Since the two most N-terminal EC repeats of protocadherin 15 interact with those of cadherin 23, the mutation has been shown to block binding of these proteins, resulting in a loss of hair bundle integrity. Specifically, *noddy* mice lack tip links and the ability to open mechanotransduction channels (Geng et al., 2013).

Zebrafish contain two genes closely related to the human *PCDH15* gene, *pcdh15a* and *pcdh15b*, which are responsible for the morphology and function of the ear and eye (Seiler et al., 2005; Maeda et al., 2017). Seiler et al. (2005) showed that mutations in *pcdh15a* result in vestibular dysfunction and deafness, while vision remains unaffected, and vice versa in the *pcdh15b* gene. It is likely that these genes are differentially expressed in these organs, reflecting the different phenotypes (Seiler et al., 2005). There are two isoforms of *pcdh15a* in zebrafish, that correspond to the human A and C isoforms, both of which are densely localized at the tip of the HC stereocilia, supporting that protocadherin 15a is a component of the HC tip links (Maeda et al., 2014, 2017).

#### Usher Type 1G: SANS

The SANS (scaffold protein containing ankyrin repeats and SAM domain) protein plays a critical role in the formation of the USH1 multiprotein complex with harmonin, myosin VIIa, and cadherin 23 (Weil et al., 2003). In mice, SANS expression has been localized to the apical region of HC stereocilia. SANS localizes to the short and middle-row stereocilia of HC and interacts with both of the tip link proteins, cadherin 23, and protocadherin 15. Therefore, SANS is predicted to be involved in a multiprotein complex localized at the lower tip link density (Caberlotto et al., 2011).

##### Structure

*SANS* contains 1,380 bp and encodes a 461 aa protein (Weil et al., 2003). The SANS protein contains three N-terminal ankyrin-like domains (ANK 1, 2, and 3), a central region (CENT), followed by a sterile alpha motif (SAM) and a tripeptide, class I PBM at the C-terminal end of the protein (Weil et al., 2003). The class I PBM of SANS interacts with the PDZ1 and PDZ2 domains of the USH2D protein, whirlin (van Wijk et al., 2006). While the SAM and class I PBM of SANS bind the PDZ1 domain of harmonin to form a highly stable complex (Yan et al., 2010).

##### Mutations

Mutations in *SANS* cause Usher syndrome type 1G (USH1G), which accounts for approximately 1% of USH cases (Weil et al., 2003; Jouret et al., 2019). Mutations in *SANS* may also result in non-syndromic HL or in USH with delayed retinal degeneration (Maria Oonk et al., 2015).

##### Animal Models

*Jackson shaker* mice that model the USH1G phenotype have been generated by mutating the *Sans* gene. These mice exhibit deafness caused by degeneration of the sensory cells in the inner ear and vestibular dysfunction characterized by head tossing, circling behaviors and hyperactivity (Kitamura et al., 1992; Kikkawa et al., 2003). *Jackson shaker* mice do not display retinal abnormalities and cannot be used to study the retinal phenotype of USH1G (Kikkawa et al., 2003). Kitamura et al. (1992) undertook a comparative study on the HC structure of homozygous *Jackson shaker* mice, and observed the differences in their HC at days P10-P30. The OHC of the homozygous *Jackson shaker* mutants exhibited incomplete differentiation, and mislocalization and disfiguration of the stereocilia (Kitamura et al., 1992).

#### Usher Type 1J: CIB2

Calcium and integrin binding protein 2 (CIB2) is a 21.6 kDa protein with 187 aa and encoded by the *CIB2* gene, comprised of 6 exons (Huang et al., 2012). CIB2 is categorized as a DNA-dependent protein kinase interacting protein, and is within the CIB protein family. CIB2 is ubiquitously expressed in a wide variety of tissues, specifically within the inner ear, CIB2 is localized to the tips of IHC, OHC, supporting cell cytoplasm, and vestibular HC stereocilia, with more concentrated expression at the tips of the shorter row stereocilia (Riazuddin et al., 2012).

##### Structure

There are four members of this protein family, CIB1-4, all containing EF-hand domains that change conformation following cation binding (Seki et al., 1999; Gentry et al., 2005; Blazejczyk et al., 2009). CIB2 has three EF-hand motifs, the last two of which mediate the cation binding activity, specifically for binding Ca^2+^ and Mg^2+^ (Huang et al., 2012; Michel et al., 2017). In contrast to previous studies, Vallone et al. (2018) showed that CIB2 is unable to function as a calcium sensor under physiological conditions due to a low affinity for Ca^2+^ ions. However, they demonstrated that CIB2 does have a high affinity for Mg^2+^ under the same conditions, and is therefore much more likely to function as a magnesium sensor (Vallone et al., 2018).

##### Mutations

Mutations in *CIB2* have been associated with both non-syndromic deafness type 48 (DFNB48) and USH type 1J (USH1J), as well as congenital muscular dystrophy type 1A (Hager et al., 2008; Riazuddin et al., 2012; Patel et al., 2015a,b). A small number of mutations in *CIB2* have been associated with USH1J, including a conservative point mutation, c.192G>C (p.E64D; Riazuddin et al., 2012). The E64 residue is in the N-terminal domain and is thought to communicate with the EF3 cation binding site in the C-terminal domain (Vallone et al., 2018). Therefore, mutations at this site prohibit this inter-domain communication, impairing the cation responsiveness of CIB2. In the presence of physiological Mg^2+^, the p.E64D mutation causes CIB2 to adopt a less stable partially unfolded conformation that is more prone to aggregation (Vallone et al., 2018). Alternative splicing gives rise to four CIB2 isoforms, and disease-causing mutations in exon 4–6 affect all isoforms (Riazuddin et al., 2012; Michel et al., 2017).

There has been dispute as to whether CIB2 should be considered an USH gene at all. The p.E64D missense mutation that is thought to cause USH1J is only two amino acids away from the p.R66W missense mutation that is exclusively linked to non-syndromic deafness (Seco et al., 2016). In addition, none of the 13 patients in a multi-ethnic cohort (Booth et al., 2018) had an abnormal retinal phenotype, regardless of the mutation type, even null alleles. Lastly, while a retinal phenotype is rarely evident in mouse models of any USH type, many do exhibit some vestibular dysfunction, unlike *CIB2* knockout mice (Giese et al., 2017; Michel et al., 2017). Further examination of the p.E64D patients is required to determine whether there may be another cause for their retinal/vestibular phenotypes.

##### Animal Models

There is currently no specific animal model for USH1J, however, a number of knockout models have been established to investigate the function of CIB2 in different organ systems. Mouse models developed using CRISPR/Cas9 to target exon four with two small deletions (one 9 bp and one 8 bp segment) introducing a premature translational stop and a truncated 109 aa protein (Wang et al., 2017). Loss of CIB2 but not CIB1 affects auditory function and causes profound HL by impacting stereocilia development (Wang et al., 2017). DPOAE thresholds were elevated indicating OHCs function deficits in homozygous knockout mice compared to heterozygous mice, and subsequent experiments have shown IHC are affected to a much lesser extent and only at later timepoints (Wang et al., 2017). Other groups have shown that Cib2 knockout mice are profoundly deaf, but have no retinal or vestibular defects (Giese et al., 2017; Michel et al., 2017; Zou et al., 2017). The deafness is likely due to knockout of CIB2 abolishing MET currents in auditory HC (Giese et al., 2017; Wang et al., 2017). Additionally, knockdown of Cib2 in zebrafish highlighted its role in the correct development, maintenance, and function of IHC (Riazuddin et al., 2012).

### Usher Type 2

USH2 patients typically have moderate SNHL from birth and RP, which starts during late puberty or early adulthood (Mathur and Yang, 2015). Although, USH2 was originally characterized as having non-progressive deafness, recent studies have shown that patients may suffer from increased loss of hearing over time, indicating symptoms can be progressive (Hartel et al., 2016). Three responsible genes have been identified so far, as well as USH2B, which was originally mapped to the short arm of chromosome 3, although following molecular analysis this gene is no longer recognized as an USH loci (Hmani et al., 1999; Hmani-Aifa et al., 2009).

#### Usher Type 2A: Usherin

*USH2A* was the first USH locus identified, with mutations in this gene causing USH type 2A and accounting for approximately half of all cases of USH (Kimberling et al., 1990; Lewis et al., 1990; Jouret et al., 2019). Approximately, 80% of USH2 cases harbor mutations in the *USH2A* gene, making it the predominant causative gene for USH2, of the three genes identified (Le Quesne Stabej et al., 2012). *USH2A* is located on chromosome 1q41 and encodes the Usherin protein, which is involved in developing and maintaining neurosensory cells in both the retina and cochlea (Bhattacharya et al., 2002). Usherin is expressed within the hair bundles during postnatal development in cochlear HC and is localized to the apical inner segment recess that wraps around the connecting cilia in photoreceptor cells (Liu et al., 2007). The cytoplasmic tail of usherin interacts with the PDZ domains of harmonin and whirlin, which are associated with USH types 1C and 2D, respectively (van Wijk et al., 2004; Adato et al., 2005a). An alternatively spliced region predicted to encode 24 additional amino acids has been identified to be specific for cochlea because the sequence is highly expressed in the inner ear (van Wijk et al., 2004; Adato et al., 2005a). This exon was previously not included in the longer *USH2A* transcript (van Wijk et al., 2004).

##### Structure

**Figure 3:**
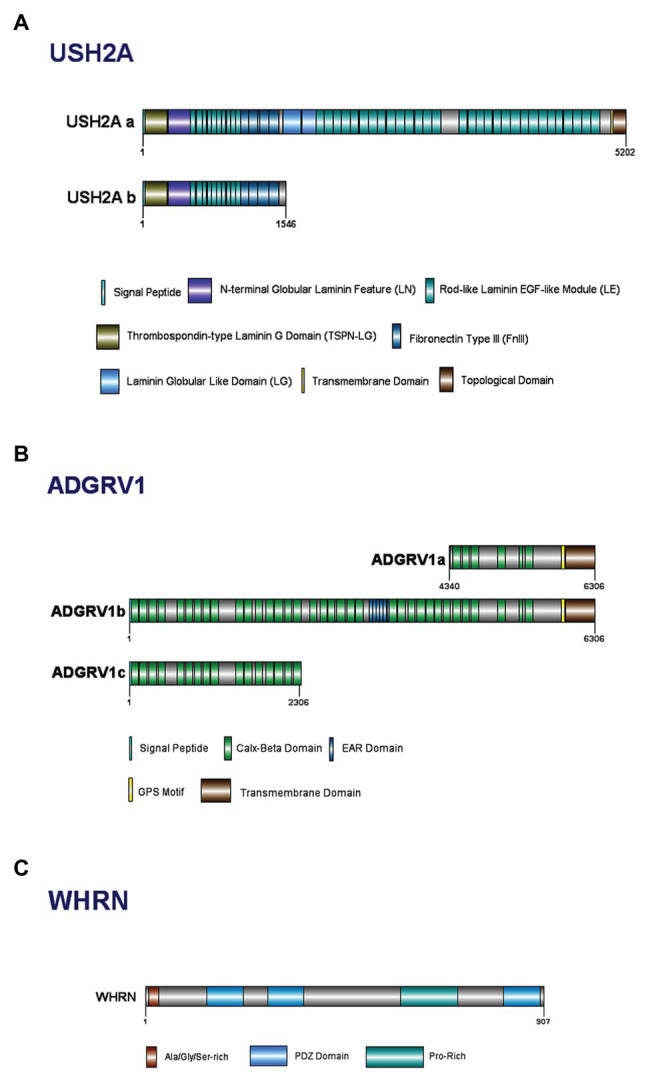
Domain structure of Usher Type II proteins and their major isoforms: **(A)** usherin; **(B)** ADGRV1; and **(C)** whirlin.

Usherin is a large protein comprised of a number of domains (Figure 3A; Eudy et al., 1998; Weston et al., 2000; Sheng and Sala, 2001; Dona et al., 2018). *USH2A* has two isoforms, A and B, with isoform B being the predominant form in retina (Figure 3A; Liu et al., 2007; Toms et al., 2015). The short isoform (A) contains 21 exons and codes for an extracellular matrix protein of ~170 kDa and 1546 aa. The long isoform (B) contains 72 exons and codes for a protein of ~580 kDa and 5202 aa, and is crucial for the development of cochlear HC (van Wijk et al., 2004; Adato et al., 2005a).

The thrombospondin-type LamG domain (TSPN-LG) has homology to the thrombospondin family of extracellular matrix proteins (Taraboletti et al., 1990; Nicosia and Tuszynski, 1994). Thrombospondin-1 has diverse functional properties, including activation of latent TGF-β1, integrin ligation and activation, as well as inhibition of both MMP-2 and MMP-9; the presence of this region in usherin suggests it may play similar roles (Sipes et al., 1999; Bein and Simons, 2000; Murphy-Ullrich and Poczatek, 2000). Following this, is the N-terminal globular laminin feature (LN module), which is required for the polymerization of laminins into the characteristic networks found in basement membranes (Bruch et al., 1989; Beck et al., 1990; Yurchenco and Cheng, 1993; Bork et al., 1996). Next, the 10 rod-like laminin-EGF-like (LE) domains comprise of repeat units of 60 aa containing eight conserved cysteines (Engel, 1989). The arrays of LE domains form rod-like tertiary structures with low flexibility and, as such, may structurally act as a rigid spacer between the amino- and carboxy-terminal domains of usherin (Beck et al., 1990). At the carboxyl terminus of usherin, there are four FN3 repeats (Yan and Liu, 2010). The FN3 repeats tend to form a series of beta-pleated sheet structures, and are associated with integrin binding molecules (Barkalow and Schwarzbauer, 1991; Bowditch et al., 1994).

##### Mutations

Usherin plays an important role in maintaining the function of the photoreceptor cells and cochlear HC. *USH2A* has a diverse mutation spectrum, including nonsense, frameshift, missense, splice site-affecting, deletion, and duplication mutations, which can all lead to hearing and vision defects in humans. Two of the most frequent mutations in *USH2A* occur on exon 13, namely c.2276C>T (p.C759F) and c.2299delG (p.E767fs), with the latter accounting for over 30% of *USH2A* mutations in an USH cohort (Le Quesne Stabej et al., 2012; Fuster-Garcia et al., 2017). Due to the high frequency of mutations on exon 13, antisense oligonucleotide treatments targeted at skipping this region of the gene are under development (Dona et al., 2018; van Wijk, 2019).

To investigate the possibility of genotype-phenotype correlations, 191 unrelated Spanish patients have been investigated for the c.2299delG and p.C759F (c.2276C>T) mutations in the USH2A gene with USH type II syndrome or non-syndromic retinal diseases (Aller et al., 2004). The c.2299delG mutation was present in patients with clinical signs of USH type II, whereas the p.C759F mutation was only associated with non-syndromic retinitis pigmentosa. Neither of these common mutations in *USH2A* have been found in patients with non-syndromic HL (Aller et al., 2004). SNHL in patients with retinitis pigmentosa may be determined by alternative splicing and the allelic variants of *USH2A* present.

##### Animal Models

*Ush2a* knockout mice have progressive degeneration of photoreceptor cells and a moderate non-progressive hearing impairment (Adato et al., 2005a). Another *Ush2a* knockout mice showed progressive photoreceptor degeneration as well as high-frequency threshold elevation (Liu et al., 2007). These *Ush2a* mutant mice models have provided information on the role of usherin in the auditory system, but limited data on the visual system. Usherin protein appears to be functional in zebrafish despite them lacking a full-length protein (Dona et al., 2018). In addition, *ush2a* knockout zebrafish have been generated successfully and these exhibit auditory abnormalities and retinal degeneration, replicating the disease phenotypes of human USH2A patients (Han et al., 2018).

#### Usher Type 2C: ADGRV1

The *ADGRV1* gene (also known as *VLGR1*, *GPR98*, or *MASS1*) is located on chromosome 5q14.3-21.3 and has been identified as the causative gene for USH type 2C (USH2C) (Hilgert, 2009a; Hilgert et al., 2009b). It encodes Adhesion G-protein-Coupled Receptor V1 (ADGRV1), a transmembrane receptor protein that is responsible for cochlear development and the formation of stereocilia ankle links (Millan et al., 2011). *ADGRV1* is one of the largest human genes, comprising 90 exons and spanning more than 600 kb of genomic DNA (Lentz and Keats, 1999, Updated 2016). ADGRV1 protein is comprised of a large number of distinct domains, and is expressed at high levels within the embryonic nervous system, in particular the ventricular zone (McMillan and White, 2004). It is also present during the development of brain and spinal cord, foetal retina and many other tissue types (Lentz and Keats, 1999, Updated 2016; Hilgert et al., 2009b).

##### Structure

The ADGRV1 protein belongs to a large N-terminal family B of seven transmembrane segment (7TM) receptors; specifically, to the subfamily with a G-protein-coupled proteolysis site for G-protein signaling (Hilgert et al., 2009b; Yang et al., 2012). ADGRV1 expresses multiple alternatively spliced variants, with three isoforms identified for humans: ADGRV1a, ADGRV1b, and ADGRV1c (Figure 3B; Yang et al., 2012). Isoform ADGRV1a starts in intron 64 of isoform ADGRV1b and contains only the last 26 exons. Containing only 1967 aa, ADGRV1a is the smallest and least abundant within the other isoforms (Lentz and Keats, 1999, Updated 2016; Hilgert et al., 2009b). Isoform ADGRV1b encodes 6306 aa and is the predominant isoform in the retina and inner ear (Lentz and Keats, 1999, Updated 2016; Zou et al., 2011). ADGRV1b has a large extracellular region, consisting of Calx-β domains, epilepsy-associated region (EAR) domains, transmembrane domains, and other exonic fragments in between the Calx-β domains (Myers et al., 2018). Isoform ADGRV1c shares the same start codon as ADGRV1b, but consists of only 31 exons (Hilgert et al., 2009b). The presence of 35 tandem-arranged CalX-β repeat domains mediate the protein-protein interactions between these two domains (McMillan et al., 2002; Yang et al., 2012). The N-terminal 29 aa of ADGRV1b are hydrophobic and may act as a signal sequence for cleavage in the mature protein (Hilgert et al., 2009b). The EAR domain consists of a repeated set of domains that form a seven-bladed propeller structure (Weston et al., 2004).

##### Mutations

Mutations in ADGRV1 account for 6.6–19% of USH2 cases (Bonnet et al., 2011; Garcia-Garcia et al., 2013). To date, more than 100 variants have been reported in ADGRV1, with about 65 and 25% being missense and small deletions mutations, repectively (Lentz and Keats, 1999, Updated 2016; Bonnet et al., 2011; Garcia-Garcia et al., 2013).

##### Animal Models

Consistent with the phenotype seen in USH2C patients, *Adgrv1/del7TM* mutant mice exhibit intact vestibular function but with HL (McMillan and White, 2004; Sadeghi et al., 2004; McGee et al., 2006). These mice have early hair bundle defects leading to a complete loss of HC in the cochlea, demonstrating the degeneration of stereocilia bundles in the OHC; however, they become profoundly deaf by postnatal day 20 (McGee et al., 2006).

#### Usher Type 2D: Whirlin

The *DFNB31* gene is located on chromosome 9q32-q34 and contains 12 exons. It encodes whirlin, a PDZ scaffold protein comprised of 907 aa and five distinct domains (van Wijk et al., 2006). The *DFNB31* gene has been reported to be responsible for non-syndromic HL type 31 (DFNB31) and USH type 2D (USH2D; Tlili et al., 2005; Aller et al., 2010). Whirlin is present at the stereociliary tips of mechanosensitive hair bundles and retinal photoreceptor cells (Belyantseva et al., 2005; Ebermann et al., 2007b). The protein isoforms of whirlin play different roles spatially and temporally in the cochlea and retina. It has been shown that in HC stereociliary bases, both full-length and C-terminal whirlin localize to the inner hair cells and vestibular hair cells and participate in the elongation process of the stereocilia in inner ears, while only C-terminal whirlin is present at the outer hair cell stereociliary tip. Full-length whirlin is the only isoform at the stereociliary base in all types of hair cells and critical for the interaction within the USH2 protein network (van Wijk et al., 2006; Mathur and Yang, 2015, 2019).

##### Structure

Whirlin has two key transcript variants in the inner ear due to alternative transcriptional start sites and/or splicing sites within the gene itself (Yang et al., 2010). The whirlin full-length isoform encodes all 12 *DFNB31* exons and contains two N-terminal PDZ domains (PDZ1 and PDZ2), a proline-rich domain and a third C-terminal PDZ domain (Figure 3C; Aller et al., 2010; Yang et al., 2010). The whirlin C-terminal isoform does not have N-terminal PDZ domains and encodes only exons 6–12; however, it retains the proline-rich region and the third PDZ domain near the C-terminus (Aller et al., 2010; Yang et al., 2010). Both isoforms are expressed in the inner ear whereas only the long isoform is expressed in the retina (Yang et al., 2010). It has been postulated that mutations at different positions of the whirlin protein cause USH2D or non-syndromic deafness (DFNB; Mathur and Yang, 2015).

##### Mutations

*DFNB31* mutations appear to be a rare cause of USH, with only two AR deafness families identified from large deafness cohort studies to date (Mburu et al., 2003; Tlili et al., 2005). The mutations reported in USH2D are either located in the C-terminal half or within the coding region specific to the long protein isoform (Mburu et al., 2003; Tlili et al., 2005; Ebermann et al., 2007b). Ebermann et al. (2007a) are the only group to report two patients being compound heterozygous for *DFNB31* mutations: one with a nonsense mutation in exon 1, resulting in a truncated protein without PDZ1 and the downstream C-terminal, whereas the other one has a splice site mutation, leading to an in-frame skipping of exon 2 and fusion of PDZ1 and PDZ2 domains. Mutations causing USH are restricted to exons 1–6, which are specific for the long isoform and plays a major role in the retina function. The short isoform expression would remain unaffected if there are mutations in exon 1 or 2 (Audo et al., 2011). Ar non-syndromics severe deafness occurs when mutations are present on both isoforms and affect PDZ3 domain, suggesting the role PDZ3 plays in photoreceptor function and maintenance (Audo et al., 2011).

##### Animal Models

The *whirler* mice have a large deletion in the middle of *Dfnb31* that causes a frameshift at p.433, leading to a premature termination (Mburu et al., 2003). As a result, the protein is truncated and does not include one of the three whirlin PDZ domains (Mburu et al., 2003). *Whirler* mice have height-ranked stereocilia and display normal orientation (Mburu et al., 2003); however, their hair bundles are consistently shorter in comparison to wild types and have a U-shaped morphology instead of the usual W-shaped bundle (Mburu et al., 2003).

### Usher Type 3

Patients with USH3 typically exhibit symptoms by mid-life including progressive SNHL and RP usually with loss of night vision (nyctalopia), constriction of visual field, as well as variable, progressive vestibular dysfunction (Kimberling et al., 2000; Fields et al., 2002). Symptoms of USH3 are quite variable with vestibular dysfunction, for example, occurring in approximately 50% of cases, and varying rates of hearing and vision symptom progression between patients. Most cases of USH3 do eventually result in profound HL even though early hearing levels are normally good (Millan et al., 2011). Type 3 has the most therapeutic potential due to the late onset of symptoms and relatively slow progression of RP (Geller et al., 2009).

#### Usher Type 3A: Clarin-1

The *CLRN1* gene is located on the long arm of chromosome 3 at position 25.1 (3q25.1), and is the causative gene for USH type 3A (USH3A; Sankila et al., 1995; Joensuu et al., 2001; Isosomppi et al., 2009). *CLRN1* is ubiquitously expressed in many tissues throughout the body, and mouse models have identified *Clrn1* expression within the HC and the spiral ganglion cells that innervate the sensory epithelium of the cochlea (Adato et al., 2002). Specifically, clarin-1 plays a role in sensory synapses, including HC and photoreceptor cells (Adato et al., 2002). Clarin-1 is also predicted to play a similar role to the USH2 proteins in the development and function of the cochlea HC bundles, but might have a distinct role in the organization of the vestibular system (Mathur and Yang, 2015).

##### Structure

USH3 is the least common form of USH, accounting for approximately 2% of all cases, although it does occur more frequently in some populations (Jouret et al., 2019). For example, USH3 is responsible for approximately 42% of cases of USH in Finland, all of which show progressive HL (Pakarinen et al., 1995; Sankila et al., 1995). There is also an increased incidence of USH3 in Ashkenazi Jewish populations in comparison to the general population, with some specific mutations being relatively common within individuals of this heritage (Fields et al., 2002; Ben-Yosef et al., 2003; Ness et al., 2003). The gene spans 1,619 bp and encodes the protein clarin-1, consisting of 232 aa and including four transmembrane domains (Adato et al., 2002; Fields et al., 2002). There are 11 alternatively spliced variants identified to date, with the main variant composed of three exons (Fields et al., 2002; Vastinsalo et al., 2011).

##### Mutations

Mutations in CLRN1 cause USH3A, likely due to the mislocalization and instability of clarin-1 protein and defective intracellular trafficking (Sankila et al., 1995; Joensuu et al., 2001; Isosomppi et al., 2009). The high incidence of USH3 in these two populations is due to a mutation founder effect in each population, specifically the c.300T>C (p.Y176X) mutation known as the Finn mayor mutation in the Finnish population, and the c.143T>C (p.N48K) mutation within Ashkenazi Jews (Joensuu et al., 2001; Ness et al., 2003; Millan et al., 2011).

##### Animal Models

*Clrn1* homozygous knockout mice show a loss of cochlea HC function, as well as a potential ribbon synapse defect in the retina (Geng et al., 2009; Tian et al., 2016). These mice exhibit disorganized HC stereocilia, circling behaviors and deterioration of their organ of Corti within the first 4 months of life (Geller et al., 2009; Geng et al., 2009, 2017). A novel USH3A mouse model, which exhibits delayed-onset progressive HL, similar to that observed in USH3 patients has been developed. The mouse model was created by exploiting the known regulatory element, Atoh1 3' enhancer, to promote transient *Clrn1* expression in HC during development. The Clrn1-KO mice were generated harboring a transgene with Clrn1-UTR cDNA (TgAC1), which was regulated by the Atoh1 3' enhancer and a β-globulin basal promoter, to control and down-regulate Clrn1 expression postnatally. In comparison to Clrn1-KO models, the KO-TgAC1 mice experience gradual HL, as shown by slowly decreasing hearing sensitivity with auditory brain stem responses (ABRs), indicating that *Clrn1* is critical in postnatal hearing preservation (Geng et al., 2017). Currently, no USH3A mouse models for the retinal phenotype exist, preventing *in vivo* studies into *CLRN1* function within the retina and hindering the development of potential therapies (Geller et al., 2009; Dinculescu et al., 2016).

#### Usher Type 3B: HARS

Histidyl-tRNA synthetase (*HARS*) codes for the protein HARS 1, a member of the class II family of aminoacyl-tRNA synthetases (O’Hanlon and Miller, 2002; Abbott et al., 2017). *HARS* synthesizes histidyl-transfer RNA (tRNA), a crucial molecule in the incorporation of histidine into proteins (Freist et al., 1999; O’Hanlon and Miller, 2002). Based on the mutation found in two patients, *HARS* was proposed to be the causative gene of USH3 (Puffenberger et al., 2012). However, these patients show signs of episodic psychosis other than USH symptoms like progressive HL, therefore it is debatable whether it could be considered as the definitive cause of USH type 3B (Puffenberger et al., 2012). To date, the localization of *HARS* in the inner ear remains unknown and the interactions of *HARS* within the USH interactome remains unclear and will require further investigations (Nolen et al., 2020).

##### Structure

The *HARS/HARS2* genes span approximately 17.4 kb and contains 13 exons (O’Hanlon and Miller, 2002). They have been mapped to the long arm of chromosome 5 at position 31.3 (5q31.3). Sharing a bi-directional promoter, *HARS2* (also known as *HARSL*) gene is aa synthetase-like gene located in a head-to-head orientation to *HARS*, with 344 bp intergenic sequence separating their open reading frame (O’Hanlon and Miller, 2002). *HARS2* bears striking homology to *HARS*, and both genes have strong structural and sequence homology over exons 3–12 (O’Hanlon and Miller, 2002). *HARS* transcripts originate from two distinct promoters: a short transcript maps 15–65 bp upstream of the ORF and a long transcript maps to a distal promoter (O’Hanlon and Miller, 2002).

##### Mutations

The c.1361A>C (p.Y454S) missense mutation in the gene is the only identified causative variant for USH3B to date. Patients with mutations in *HARS* not only experience the classical USH symptoms of progressive hearing and vision loss, but also experience episodic hallucinations or psychosis, commonly during periods of acute febrile illness (Puffenberger et al., 2012; Abbott et al., 2017). *HARS* has been found to be thermally sensitive, in that cells from patients harboring the p.Y454S mutation show reduced levels of protein synthesis at higher temperatures. Additionally, USH3B patients often exhibit delayed motor development, and have a wide-based gait or mild truncal ataxia (Abbott et al., 2017). Due to the additional psychotic and neurological symptoms in patients with this mutation, it is currently undecided whether *HARS* should be considered an USH gene, or whether it is instead the cause of a different rare syndrome or group of syndromes (Mathur and Yang, 2015).

##### Animal Models

There was a severe reduction in the number of sensory hair cells in the lateral line at 72 h post fertilization when *hars* was knocked down in embryos of a transgenic line, *Tg(pou4f3:GFP*; Waldron et al., 2019). *Hars knockdown* zebrafish embryos showed a decrease in proliferative cells and an increase in apoptotic cells (Waldron et al., 2019). Stress-related genes such as *asns*, *gpt2*, and *eif4ebp1* were activated and strongly expressed in the *hars* knockdown embryos, suggesting that *hars* knockdown inhibits cell proliferation by inducing the Amino Acid Starvation Response (Waldron et al., 2019).

## Interactions Between USH Proteins in the Inner Ear

The proteins implicated in USH are all closely related in their region of expression and function within the inner ear. Many of these proteins interact to form important structures such as stereociliary links and the MET complex. The MET channel consists of different subunits whose genes are involved in hearing impairment, namely they are transmembrane inner ear protein (TMIE), transmembrane channel like protein (TMC1), and tetraspan membrane protein of hair cell stereocilia (TMHS). Interactions of these MET channel subunits with the C-terminal domain of Protocadherin 15 occurs at the tip link in hair cells, and is essential for mechanotransduction MET machinery of hair cells (Xiong et al., 2012; Zhao et al., 2014; Beurg et al., 2015; Kurima et al., 2015).

**Figure 4:**
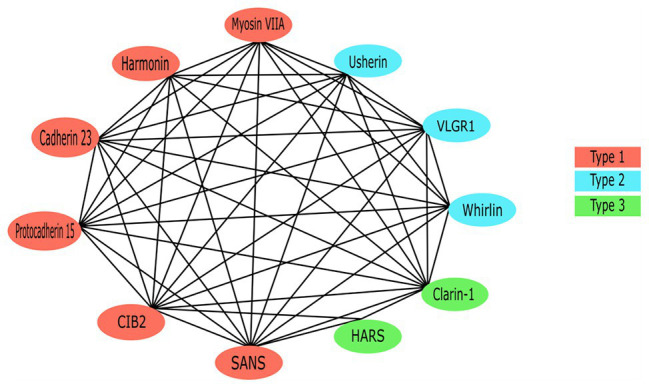
The Usher protein interaction network. All previously reported interactions among Usher proteins have been indicated.

Each of the USH proteins are known or predicted to interact with several of the other USH protein members (Figure 4). One of the most important pairs of proteins in maintaining stereociliary bundle formation is cadherin 23 and protocadherin 15. These proteins interact with themselves and their extracellular domain homodimers interact *in trans* with each other at their N-termini (Alagramam et al., 2011). Cadherin 23 and protocadherin 15, localized to the upper and lower parts of the tip link, respectively, form the connections between the HC stereocilia that are critical for stabilizing the stereocilia bundle structure. Mutations affecting either of these proteins can prevent their ability to interact and thereby cause critical defects in tip link formation (Kazmierczak et al., 2007). The tip links are also responsible for transmitting force to the MET complex *via* interactions with MET complex proteins (Maeda et al., 2014, 2017). Components of the MET (or upper tip link density) complex include myosin VIIa, harmonin, and SANS, which localize to the region on the taller stereocilium where the tip link anchors and interact in a large complex with cadherin 23 to regulate MET and tip link tension (Figure 1; Adato et al., 2005b; Grati and Kachar, 2011; Cunningham and Muller, 2019). Cadherin 23 also frequently complexes with myosin-1c and harmonin, which helps to shape and stabilize the stereocilia of the hair bundles (Liu et al., 2012). These proteins act as a bridge between cadherin 23 and the cytoskeletal actin core of stereocilium through binding of the PDZ domains and anchors cadherin 23 intracellularly to the actin filaments (Boeda et al., 2001, 2002; Adato et al., 2005a).

Due to the role it plays as a scaffold protein, harmonin is considered to be at the core of the USH protein interactome (Reiners et al., 2005a). Specifically, the PDZ1 and/or PDZ3 domains, and N-terminal domains of harmonin form a supramodule that binds the SAM domain on SANS with high affinity, forming a stable complex between the two scaffold proteins (Adato et al., 2005b; Yan et al., 2010). The complex binds the tail-region of myosin VIIa to form the upper tip link density or motor complex, which anchors the tip link to the taller stereocilia *via* binding of the CD of cadherin 23 proteins (Boeda et al., 2002; Adato et al., 2005b; Bahloul et al., 2010; Yan et al., 2010). Known USH-causing missense mutations in both of the genes has been shown to adversely affect the formation of this harmonin/SANS complex and therefore supports that the structure plays a role in normal hearing function (Yan et al., 2010). Additionally, the cytoplasmic tail of protocadherin 15 has been shown to bind the PDZ2 domain of harmonin *in vitro* (Adato et al., 2005b; Senften et al., 2006). Co-localization of USH2 proteins and harmonin in both the HC and retina may indicate the inclusion of *USH2A* and *ADGRV1* products in the USH-protein supramolecular network (Reiners et al., 2005b). Both usherin and ADGRV1 contain PBM in their C-termini, which bind the PDZ1 domain of harmonin (Reiners et al., 2005b). As well as binding other USH proteins, *in vitro* assays have identified that the second coiled-coil domain of harmonin isoform b is able to bind the PDZ1 and PDZ2 regions of all other harmonin isoforms (Adato et al., 2005b).

Another central protein is myosin VIIa due to its role in the facilitation of USH protein trafficking *via* binding of ATP and the actin-core of stereocilia (Udovichenko et al., 2002; Woolner and Bement, 2009). Specifically, the C-terminal MyTH4-FERM domain of myosin VIIa binds to the PDZ domains of harmonin, whirlin as well as the USH modifier, PDZD7 (Li et al., 2017). In the absence of myosin VIIa, harmonin isoform b is not detectable along the stereocilia, implicating a dependence on the motor protein for transport of this isoform to its correct subcellular location, which is not seen in isoforms a or c (Boeda et al., 2002). The CD of protocadherin 15 bind myosin VIIa, suggesting that the two proteins interact in some way to facilitate the development and regulation of HC integrity, roles which both proteins are associated with (Senften et al., 2006). Among the USH proteins, CIB2 only interacts with myosin VIIa and whirlin (Riazuddin et al., 2012). While this interaction is not necessary for localization of CIB2 to the stereocilia tips (Riazuddin et al., 2012), knockout of CIB2 results in mislocalization of whirlin (Michel et al., 2017). Additionally, the PDZ1 and PDZ2 domains of whirlin interact with the class I PBM of SANS (van Wijk et al., 2006). SANS interacts with both tip link proteins, cadherin 23 and protocadherin 15, therefore is predicted to be involved in a multiprotein complex localized at the lower tip link density (Figure 1; Caberlotto et al., 2011).

The USH2 proteins usherin, ADGRV1, and whirlin interact with one another to form a multiprotein complex called the ankle link (or USH2) complex within the cytoplasmic region of ankle links (Mathur and Yang, 2015). The myosin VIIa protein is predicted to transport all three of the USH2 proteins to the base of the stereocilia to form the transient ankle link (Michalski et al., 2007). Ankle links are the thin fibers that connect the bases of neighboring stereocilia and only exist during development (Michalski et al., 2007). Defects in the ankle link complex result in disorganization of the stereocilia bundle. As the correct organization of the stereocilia hair bundle is critical for accurate processing of sound, mutations in the genes coding for these USH2 proteins could cause SNHL through the disruption of the ankle link structure during development (Michalski et al., 2007). The phenotype in USH2A patients with defective usherin is remarkably similar to that seen in USH2C patients with inactivated ADGRV1, therefore suggesting the co-localization of ADGRV1 and usherin in specific sub-cellular compartments (Weston et al., 2004). The long whirlin isoform is responsible for the formation of the USH2 protein complex *in vivo*; therefore, when whirlin is disrupted the normal cellular localization of usherin and ADGRV1 are abolished, resulting in hearing and visual defects (Yang et al., 2010). The latter, is predicted to be the result of whirlin interacting with usherin, as well as USH1 proteins harmonin and SANS, to stabilize the centrosome-cilium interface region of the photoreceptor cells (Chen et al., 2014; Sorusch et al., 2017).

Clarin-1 interactions are less studied than other USH proteins, although the protein is predicted to interact with myosin VIIa and play a role in regulation of the actin cytoskeleton of the stereocilia (Adato et al., 1999; Tian et al., 2009). Evidence suggests that *CLRN1* and *MYO7A* products may be involved in a synergistic interaction, with symptoms of USH3 being more severe in the presence of a single mutated *MYO7A* allele (Adato et al., 1999). Therefore, patients harboring a single pathogenic *MYO7A* mutation, which would not usually be disease causing in isolation, may present with more severe USH symptoms if they also carry two defective *CLRN1* alleles (Adato et al., 1999). Additionally, clarin-1 is associated with protocadherin 15, with the USH3A protein predicted to increase the efficiency of assembly and localization of the USH proteins that form the mechanotransduction machinery, through modulation of vesicle recycling (Ogun and Zallocchi, 2014). Specifically, the C-terminal tail of clarin-1 is known to interact with protocadherin 15, since normal localization and function of protocadherin 15 is inhibited in mouse models expressing truncated clarin-1 (Ogun and Zallocchi, 2014).

## Pdzd7: an Usher Modifier

*PDZD7* encodes a PDZ domain-containing scaffold protein, which is responsible for the organization of the USH2 interactome (Zou et al., 2014; Du et al., 2018). PDZD7 is considered an USH modifier as it interacts with both USH1 and USH2 proteins, including myosin VIIa, usherin, ADGRV1 and whirlin (Ebermann et al., 2010; Zou et al., 2014; Du et al., 2018). As a result, mutations in *PDZD7* can cause non-syndromic recessive hearing impairment and also interrupt organization of cells and disrupt the MET process, causing SNHL and deafness as seen in USH (Du et al., 2018). The formation of the USH2 quaternary protein complex relies on the heterodimerization between PDZD7 and whirlin and a subsequent dynamic interplay between USH proteins *via* their multiple PDZ domains (Chen et al., 2014). Progressive retinal cell death and a reduction in ADGRV1 localization in the connecting cilia in photoreceptor cells were observed in a *PDZD7*-knockdown zebrafish, which has an USH-like phenotype. In addition, *PDZD7*-knockout mice exhibit a loss in stereocilia bundle architecture at the ankle-link region, resulting in the attenuation of MET energy (Zou et al., 2014). As such, *PDZD7* mutations can cause symptoms of USH *via* disruptions in its interactions with other proteins rather than through its own specific roles.

## Therapeutic Approaches

Unfortunately, despite significant advances in the field, there is still no cure for USH, regardless of the subtype or specific causative gene mutation. Several treatment options are available to help patients manage the symptoms of USH, particularly those of HL. Treatments for the visual and balance symptoms are more limited, with most strategies simply providing support to assist patients in completing basic living tasks. The current treatment for all types of HL, including USH, is auditory rehabilitation using hearing aid devices or cochlear implants. These technologies can significantly aid in communication, yet they are still unable to mimic the quality of natural hearing and, more importantly, they do not treat the underlying cause of the HL. Currently, there are a range of new therapeutic strategies being developed that utilize new technologies such as gene editing and cell based therapies, with some clinical trials currently underway.

### Viral-Based Gene Replacement Therapy

One therapeutic approach for genetic disorders including USH is to replace the mutated gene with a wildtype copy of the gene, allowing functional protein to be produced. A popular option for delivering replacement genes is using viral vectors, such as lentivirus and adeno-associated virus (AAV). Local delivery of rAAV2/8 containing a replacement *Sans* gene into the inner ear completely restored vestibular function and hearing in *Sans knockout* mice (Emptoz et al., 2017). Although AAVs have a smaller packaging capacity than lentiviral vectors, AAV-mediated delivery of full-length *MYO7A* cDNA has been effective both *in vivo* and *in vitro*, resulting in wildtype expression levels (Allocca et al., 2008; Lopes et al., 2013; Dyka et al., 2014; Trapani et al., 2014). As AAV has limited genetic capacity, many gene sequences are too large to fit into a single vector (Akil, 2020). A full length *MYO7A* cDNA was successfully reconstituted in an AAV post-infection, however the vectors were in a heterogenous, fragmented genome (“fAAV”) form, with low efficiency (Lopes et al., 2013). To address this, Dyka et al. (2014) used an overlapping approach with dual vectors sharing a central part of the *MYO7A* cDNA sequence, leading to the expression of full length *MYO7A* transcript *in vitro* and *in vivo* with equal and higher efficiency than fAAV. Several research groups have used dual or multi AAV systems to overcome this limitation in USH studies, including oversized transgene constructs. However, most studies have reported very low levels of protein expression (Jaijo et al., 2007; Allocca et al., 2008; Alagramam et al., 2011; Dyka et al., 2014; Trapani et al., 2014; Maddalena et al., 2018).

### Genome Editing-Based Therapy

#### Zinc-Finger Nucleases and CRISPR/Cas9

Genome editing-based therapy is another promising approach for treating USH as it attempts to correct the mutation *in vivo* in a site-specific manner, while preserving endogenous regulation of the repaired gene. Zinc-finger nucleases (ZFN) are chimeric proteins generated by fusing a zinc-finger DNA-binding domain (ZF domains) to a DNA-cleavage domain (Durai et al., 2005). ZFN was applied to target a nonsense mutation (p.R31X) in *Ush1c* cell line, leading to recovered harmonin protein expression, with no apparent off-target effects (Overlack et al., 2012). While ZFN seem like a powerful tool, the clustered regularly interspaced palindromic repeats (CRISPR) and CRISPR-associated protein 9 (Cas9) system is now the most advanced and widely accepted methodology due to its simplicity, robustness, and high efficiency (Mishra et al., 2018).

Clustered regularly interspaced palindromic repeats/CRISPR-associated protein 9 was used to successfully correct one of the most prevalent mutations in *USH2A* (c.2299delG) in patient-derived fibroblasts (Fuster-Garcia et al., 2017). A CRISPR/Cas9 approach has also been used on patient-derived induced pluripotent stem cell (iPSC) harboring mutation in *USH2A* or *MYO7A* (Tang et al., 2016; Sanjurjo-Soriano et al., 2019). In these studies, the iPSC were successfully corrected with high efficacy and specificity, with no observed off-target mutagenesis (Tang et al., 2016). Additionally, the corrected iPSC retained their pluripotency and were genetically stable (Sanjurjo-Soriano et al., 2019).

Allergan and Editas Medicine have recently launched the Brilliance clinical trial (NCT03872479) for Leber congenital amaurosis (LCA) 10 patients with an intronic mutation in *CEP290* gene. EDIT-101 (AGN-151587) from Editas is designed to locate and remove the mutated *CEP290 via* based on non-homologous end joining (Maeder et al., 2019). Published results showed sub-retinal delivery of EDIT-101 achieved rapid, productive and sustained *CEP290* gene editing in *CEP290* mice and somatic primate cells *in vivo* (Maeder et al., 2019). Concurrently, EDIT-102 is being developed to target USH2A using the same proprietary enzyme, vector, promoters, and route of delivery as EDIT-101. Since *USH2A* mutations can affect cell receptors in the same way as *CEP290* mutations, the USH2A product is expected to benefit from EDIT-101’s therapeutic pipeline.

### Drug Therapy

#### Antisense Oligonucleotides

Antisense oligonucleotides (ASO) are short, synthetic, single-stranded oligodeoxynucleotides that are complementary to the mRNA target (Rinaldi and Wood, 2018). ASO have been used to correct cryptic splicing in *Ush1c*.216A knock-in mice (Lentz et al., 2013). This partial correction by ASO increases harmonin expression, improves the morphology and structure of stereocilia, leading to a rescue of auditory and vestibular responses in Ush1c mice. This shows that early-treated mice respond better due to an age threshold for effective drug delivery (Lentz et al., 2013; Vijayakumar et al., 2017; Donaldson et al., 2018). Similarly, Ponnath et al. (2018) reported restoration of OHC and IHC in Ush1c mice by ASO-29 treatment.

Two designed ASO successfully targeted a pseudo-exon-causing *USH2A* mutation, increasing the amount of correctly spliced transcript in *USH2A* patient-derived fibroblasts (Slijkerman et al., 2016). A novel drug, QR-421a (ProQR Therapeutics) demonstrated a successful “exon skip” in an *Ush2a* mutant zebrafish model, leading to restored Usherin expression and electroretinogram (ERG) recordings (Dona et al., 2018; van Diepen et al., 2019). QR-421a is currently in clinical trial (Stellar; NCT03780257), with preliminary results reported in April 2020.^1^

#### Reducing Endoplasmic Reticulum Stress

The endoplasmic reticulum (ER) is responsible for processing mRNA into protein and appropriately folding those proteins (Szegezdi et al., 2006). Importantly, the ER is also the site at which several USH proteins complex before trafficking as a group into their final position (Blanco-Sanchez et al., 2014). A mutation in a single USH gene can prevent the protein complexes from forming and lead to an accumulation of multiple USH proteins in the ER, which causes ER stress and, ultimately, apoptosis (Blanco-Sanchez et al., 2014). Three proteins in particular – harmonin, cadherin 23 and myosin VIIa – have been shown to co-localize in the ER and preassemble as a complex prior to trafficking to the stereocilia (Blanco-Sanchez et al., 2014). Treatment of *erlong* (*erl*) mice, *Cdh23* mutant mice carrying a missense mutation with the small molecular compound Salubrinal®, an ER stress inhibitor, caused a reduction in auditory brainstem response thresholds and higher distortion product otoacoustic emission (DPOAE) amplitudes (Han et al., 2012; Hu et al., 2016). Taken together, these findings suggest that inhibition of ER stress may slow or prevent inner ear HC death and subsequent HL in USH patients (Szegezdi et al., 2006).

#### Improving the Stability of USH Proteins

The most common causative mutation for USH type III in Ashkenazi Jews of European and North American descent is the c.144T>G (p.N48K) mutation in *CLRN1* (Fields et al., 2002; Ness et al., 2003). This mutation is thought to adversely affect the folding and stability of the clarin-1 protein, ultimately causing progressive vision and HL (Tian et al., 2009). A novel therapeutic approach is to improve the stability of the mutant clarin-1 protein, thereby allowing it to correctly localize to the plasma membrane and restore function. An intensive high-throughput screening approach identified 48 candidate small molecules that act to stabilize the CLRN1^N48K^ *in vitro* (Alagramam et al., 2016). A secondary screen of these candidates for proteasome activity found three small molecules that showed clear evidence of specific stabilization of CLRN1^N48K^. Final validation by immunoblotting narrowed this down to a single candidate, O03, which was effective in the two cell lines tested. Subsequent testing confirmed that the increase in cellular CLRN1^N48K^ following O03 treatment was due to increased stability of the mutant protein (Alagramam et al., 2016). A synthetic modification of O03, BF844, improved the potency and pharmacokinetics of the compound. When applied before or during the early stages of HL, BF844 effectively crossed the blood-labyrinth barrier and mitigated HL in a mouse model (Alagramam et al., 2016). This represents a potential therapeutic candidate for USH3A patients with this mutation that could slow or prevent HL, if applied prior to its onset.

### Cell-Based Therapy

#### Stem Cells and Three-Dimensional Organoids

Given the capacity of pluripotent stem cells (PSCs) to self-renew and differentiate into many cell types, they are now widely used in regenerative medicine (Okano and Kelley, 2012). The stem cells commonly used are embryonic stem cells (ESCs), adult stem cells (ASCs), and iPSC (Dufner-Almeida et al., 2019). Oshima et al. (2010) adapted the protocol and promoted the differentiation of mouse ESCs and iPSC into mechanosensitive HC-like cells.

Pluripotent stem cells can form embryoid bodies *in vitro* which are three dimensional (3D) aggregates capable of differentiating into specific lineages (Okano and Kelley, 2012; Omole and Fakoya, 2018), including retinal (Nakano et al., 2012; Phillips et al., 2012; Zhong et al., 2014; Mellough et al., 2015, 2019) and inner ear organoids (Koehler and Hashino, 2014; Liu et al., 2016; Longworth-Mills et al., 2016; Nie et al., 2017). The first inner ear organoid system was developed by Koehler et al. using mouse ESCs, which was later adapted to use human ESCs or iPSC (Koehler et al., 2017). Several groups have published protocols inducing inner ear organoids from aggregates of mouse ESCs (Koehler and Hashino, 2014; Liu et al., 2016; Longworth-Mills et al., 2016; Nie et al., 2017) and human PSCs (Jeong et al., 2018). Single-cell electrophysiology tests identified functional vestibular hair cells in inner ear organoids (Jeong et al., 2018). Numerous improved protocols have been described on including the modulation of signaling pathways, like Wnt, Sonic Hedgehog, and Fgf (Lahlou et al., 2018), addition of extracellular matrix scaffolds (e.g., Matrigel; Koehler et al., 2013, 2017); and addition of bone morphogenetic protein (BMP), transforming growth factor beta (TGFβ), and Wnt antagonists (Zhou et al., 2015). These protocols have been discussed in depth in a recent review (Tang et al., 2020). The PSCs and organoid technology are ideal for creating patient-specific disease models for USH, with these *in vitro* models possessing an identical genomic profile of the patient, offering opportunities for personalized medicine (Johnson and Hockemeyer, 2015; Yilmaz and Benvenisty, 2019).

#### Hair Cell Regeneration

Since hearing loss commonly occurs as a result of inner ear hair cell damage and since hair cells are unable to regenerate spontaneously (i.e., quiescent), promoting the regeneration of these hair cells is a key therapeutic goal. Cellular quiescence occurs *via* inhibition of cyclin/cyclin-dependent kinase (CDK) and hypophosphorylation of retinoblastoma protein (Polyak et al., 1994). CDK inhibitors such as p27Kip1 are expressed in the cochlea at a level sufficient to promote quiescence of the hair cells (Mantela et al., 2005; Laine et al., 2007; Walters et al., 2014). Deletion of p27Kip1 in transgenic mice resulted in proliferation of supporting cells and regeneration of hair cells (Chen and Segil, 1999; Lowenheim et al., 1999; Kil et al., 2011). Hair cell-specific conditional deletion of p27Kip1 in neonatal mice resulted in proliferation and improved survival of hair cells without any adverse effects on hearing (Walters et al., 2014). In response to the knockout of p27Kip1, IHC were more proliferative than OHC (Walters et al., 2014). These findings suggest that p27Kip1 could represent a good therapeutic target to minimize cell death and promote hair cell regeneration in the cochlea. As such, Sound Pharmaceuticals is conducting a phase 1/2 trial (NCT02819856) of SPI-5557, a drug that inhibits p27Kip1, aiming to regenerating cochlea hair cells (Jones, 2018).

Atoh1 (also known as Math1 for “mouse atoh1” and HATH1 for “human atoh1”), a basic helix-loop-helix transcription factor, was first identified to be essential in the generation of inner ear hair cells, and overexpression of *Atoh1* enhanced hair cell regeneration (Ben-Arie et al., 1997; Bermingham et al., 1999). Atoh1 has since been in use as a prime candidate in hair cell regeneration research, with varying success rates across published studies. Guinea pigs treated with *ATOH1* gene therapy showed a significant increase in the number of hair cells (Atkinson et al., 2014). Overall, these guinea pigs did not have complete recovery of hearing, suggesting that ATOH1 gene therapy alone is unable to convert non-sensory cells into hair cells, nor capable of rescuing the phenotype of surviving hair cells (Atkinson et al., 2014).

### Limitations and Considerations for USH Therapies

Given that USH is a syndromic condition affecting multiple senses, it is important to note that current therapeutic trials generally focus on one sense rather than across the entire syndrome. The onset of visual symptoms is later than the onset of audiological symptoms in all USH sybtypes (Mathur and Yang, 2015). Upon identification of HL, genetic testing would indicate if a mutation is present in the known USH-related genes, which provides information on the USH subtype and the disease progression. It follows then that therapeutics could be delivered to a patient before their vision deteriorates and maintaining important protein structures and complexes is a much easier task than restoring them. Delivery of drugs into the inner ear is notoriously difficult. Systemic delivery carries the highest risk for off target effects and, regardless, is hampered by the blood-labyrinth barrier, which is the physiological barrier between the peripheral blood and the inner ear fluids (Hao and Li, 2019; Nyberg et al., 2019). Direct injection into the scala media would result in the highest bioavailability and benefit, however this requires a costly and high risk surgery (Hao and Li, 2019; Nyberg et al., 2019).

There are several barriers to overcome in order to achieve successful gene therapy, including the likelihood of a host immune response being triggered and directed against the vector component or the transgene product, or both, as these vector systems are administered directly to the patient (Williams et al., 2017). Another uncertainty is whether the transgenes have long-term expression and the same functional response as a healthy gene. As for genome editing approaches, there are a number of disadvantageous consequences to be considered – off-target mutagenesis, mosaicism, complex rearrangements, on-site damage, and biallelic modification (Araki et al., 2014). These technical risks can be reduced and addressed by experimental design; for example, designing unique genomic target sequences and evaluate off-target risks with bioinformatics tools, but there are still too many unknowns regarding the mechanisms of DNA repair at this stage (Araki et al., 2014). It is challenging to determine the therapeutic vector dose and the route of administration, as it is not well characterized at this stage. Many strategies have been conducted to overcome human immune responses in a systemic gene transfer, such as delivering at a different site or administering immunosuppressive drugs to prevent or block T-cell responses toward foreign antigens (Sack and Herzog, 2009), much more remains to be done to better understand the efficacy and safety implications of long-term risk of gene therapy administration.

Although stem cell-based therapy is popular, there are several challenges and unanticipated risks to be evaluated before it could be applied clinically. The major concern is the similarities between stem cells and cancer cells, such as the ability to self-renew, the potency of stem cells (pluripotent or multipotent), indefinite growth, and high proliferation rate (Li and Neaves, 2006; Werbowetski-Ogilvie et al., 2009; Herberts et al., 2011). The first point is the survival rate and period of the transplanted cells after their insertion into the patients’ cochlea, without interfering the normal hearing. Alongside the risk of tumorigenesis, potential stem cell migration to inappropriate sites and immune rejection of transplanted stem cells must be considered. Other risk factors include the type of stem cells used, their procurement and culturing history, the level of manipulation and administration site associated with stem cell-based therapy and medicinal products (Herberts et al., 2011).

## Future Outlooks

Although USH genes have been mapped and many corresponding pathological mutations identified, the syndrome remains incurable. USH proteins interact in large multiprotein complexes in the inner ear and retina, and are responsible for the trafficking, scaffolding, and signaling of proteins involved in the development and maintenance of the sensory cells of these organs. Within the cochlea, USH proteins play critical roles in the correct organization and function of the HC stereocilia that conduct soundwaves to the brain. A deeper understanding of these complex USH protein networks, including their functions and the downstream effects of protein mutations in the inner ear sensory cells, is critical for understanding USH. Research into the genotype–phenotype associations of USH gene mutations has highlighted the importance of accurate molecular diagnosis for patients, since the nature of the mutation can greatly influence the prognosis (Toms et al., 2015; Bonnet et al., 2016).

Interestingly, all current potential treatments are only targeted at one of the groups of symptoms (HL, vision loss, or balance problems), none have looked at tackling the syndrome as a whole. Currently, a majority of the USH treatments under development are targeted at specific, high-frequency mutations, further emphasizing the need for genetic screening (Toms et al., 2015; Jouret et al., 2019). Meta-analysis of USH-causing mutations have been successful in highlighting the frequency and location of pathological mutations, including hotspot and founder mutations (Jouret et al., 2019).

Significant progress has also been made into the characterization of USH pathogenesis through animal models. Recent studies have begun to elucidate the complex functions and interactions between USH proteins, although there remains a number of unknowns regarding the molecular mechanisms controlling these processes, as well as possible gene interactions. Inner ear organoids generated from human cells may hold the key to the *in vitro* analysis of human USH genes and proteins. Particularly given that several of the USH genes are responsible for early development, which would be mimicked by the organoids (Liu et al., 2016; Koehler et al., 2017; Jeong et al., 2018). Presently, a number of studies are underway to identify potential USH treatments, including ASO therapy, CRISPR/Cas9 genome editing, translational read-through pharmaceutical drugs, and AAV or lentiviral-mediated therapies. Clinical trials focused on applying these techniques to USH in the hopes of developing a cure, are still underway.

## Author Contributions

MA, RD, and EW designed the study. MW, AF, ZN, and XK conducted the research and literature search. MW, AF, ZN, and EW prepared the manuscript. All authors contributed to the article and approved the submitted version.

### Conflict of Interest

The authors declare that the research was conducted in the absence of any commercial or financial relationships that could be construed as a potential conflict of interest.

## Footnotes

^1^https://ir.proqr.com/news-releases/news-release-details/proqr-announces-positive-findings-interim-analysis-phase-12
